# Controlling of CSFV in European wild boar using oral vaccination: a review

**DOI:** 10.3389/fmicb.2015.01141

**Published:** 2015-10-23

**Authors:** Sophie Rossi, Christoph Staubach, Sandra Blome, Vittorio Guberti, Hans-Hermann Thulke, Ad Vos, Frank Koenen, Marie-Frédérique Le Potier

**Affiliations:** ^1^Unité Sanitaire de la Faune, Office National de la Chasse et de la Faune SauvageGap, France; ^2^Friedrich-Loeﬄer-Institut, Federal Research Institute for Animal HealthGreifswald, Germany; ^3^Instituto Superiore per la Protezione e la Ricerca AmbientaleOzzano dell′Emilia, Italy; ^4^Department of Ecological Modelling, Helmholtz Centre for Environmental Research-UFZLeipzig, Germany; ^5^Development Vaccines Technologies, IDT Biologika GmbHDessau-Rosslau, Germany; ^6^Operational Direction Interactions and Surveillance, Centrum voor Onderzoek in Diergeneeskunde en Agrochemie-Centre d’Etude et de Recherches Vétérinaires et AgrochimiquesUkkel, Belgium; ^7^Laboratoire de Ploufragan/Plouzané, Unité Virologie Immunologie Porcines, AnsesPloufragan, France

**Keywords:** *Pestivirus*, wildlife, diseases, management, surveillance, *Sus scrofa*

## Abstract

Classical swine fever (CSF) is among the most detrimental diseases for the swine industry worldwide. Infected wild boar populations can play a crucial role in CSF epidemiology and controlling wild reservoirs is of utmost importance for preventing domestic outbreaks. Oral mass vaccination (OMV) has been implemented to control CSF in wild boars and limit the spill over to domestic pigs. This retrospective overview of vaccination experiences illustrates the potential for that option. The C-strain live vaccine was confirmed to be highly efficacious and palatable baits were developed for oral delivery in free ranging wild boars. The first field trials were performed in Germany in the 1990’s and allowed deploying oral baits at a large scale. The delivery process was further improved during the 2000’s among different European countries. Optimal deployment has to be early regarding disease emergence and correctly designed regarding the landscape structure and the natural food sources that can compete with oral baits. OMV deployment is also highly dependent on a local veterinary support working closely with hunters, wildlife and forestry agencies. Vaccination has been the most efficient strategy for CSF control in free ranging wild boar when vaccination is wide spread and lasting for a sufficient period of time. Alternative disease control strategies such as intensified hunting or creating physical boundaries such as fences have been, in contrast, seldom satisfactory and reliable. However, monitoring outbreaks has been challenging during and after vaccination deployment since OMV results in a low probability to detect virus-positive animals and the live-vaccine currently available does not allow serological differentiation of infected from vaccinated animals. The development of a new marker vaccine and companion test is thus a promising option for better monitoring outbreaks during OMV deployment as well as help to better determine when to stop vaccination efforts. After rabies in red fox, the use of OMV against CSF in European wild boar can be considered as a second example of successful disease control in wildlife. The 30 years of disease control experience included in this review may provide options for improving future disease management within wild populations.

## Introduction

Classical swine fever (CSF) is a major threat to commercial pig production worldwide (Edwards et al., 2000). This multi-systemic disease can affect both domestic pigs and wild boar such that outbreaks among wild boar can significantly impact commercial pig farms. CSF outbreaks among wild boars present a constant threat of introduction into domestic pigs. In Germany during the 1990’s, approximately two thirds of primary outbreaks among domestic pigs were attributed to direct or indirect contact with CSF infected wild boar (Fritzemeier et al., 2000).

Generally, control of wildlife reservoirs is a significant challenge (Delahay et al., 2009; Gortázar et al., 2015). To combat infectious diseases, vaccination is often used to decrease the proportion of susceptible animals in a population below a threshold needed for disease maintenance among that population (Rupprecht et al., 2003; Blancou et al., 2009).

Different vaccination approaches for wild boar have been developed and tested, some of them directly under field conditions (e.g., lyophilized vaccines in Russia), others under experimental conditions (Kaden et al., 2000). Under experimental conditions, live attenuated vaccines showed high efficacy and complete safety upon oral immunization of individual animals (Kaden et al., 2000). To deliver the vaccines on a larger scale, oral bait formulations were subsequently developed and tested by Kaden et al. (2000) during the 1990’s. These baits were suitable for oral mass vaccination (OMV) and in the following years the approach was considered as a satisfactory option for improving CSF virus (CSFV) control in wild boar in Western Europe (EFSA, 2008; von Rüden et al., 2008; Rossi et al., 2010).

However, upon implementation of large scale oral CSF vaccination, it was discovered that the vaccination process and design need further improvement and was subsequently revisited. As a consequence, adaptations were introduced in all areas of CSF control in wild boar including the baiting strategy, population management, and surveillance design (Rossi et al., 2014). This review addresses and summarizes multiple aspects of oral vaccination of wild boar including its successes and failures, its drawbacks and advantages.

## Vaccination Tools

### Vaccines

Several CSFV vaccines are available and have been used successfully to control the disease in multiple countries worldwide (van Oirschot, 2003; Greiser-Wilke and Moennig, 2004; Blome et al., 2006; Luo et al., 2014). The most widely used vaccines are conventional live attenuated vaccines including the well-known lapinised “Chinese” C-strain or its derivatives, and the Thiverval strain. These vaccines have shown outstanding efficacy and safety, but do not allow serological differentiation of infected animals from vaccinated ones; for this reason, vaccinated animals are subject to trade restrictions. To overcome these limitations, marker vaccines have been developed based on different vector platforms and expression systems (for review see Beer et al., 2007; Dong and Chen, 2007; Blome et al., 2013). These approaches allow differentiation for field detection of virus infection versus vaccination (DIVA; van Oirschot, 2003; Leifer et al., 2009).

#### Live Attenuated Vaccines

These traditional live attenuated vaccines have been used worldwide in eradication campaigns both intramuscularly (IM) in domestic pigs and in oral bait formulations in wild boar (Kaden and Lange, 2001). IM application of these vaccines confers protection a few days after immunization (van Oirschot, 2003), before neutralizing antibodies are detected. Antibody detection is typically possible within 2 weeks after vaccination (Kaden and Lange, 2001; Vandeputte et al., 2001). Upon oral immunization, protection is usually conferred within 2 weeks or less (Kaden and Lange, 2001; Blome et al., 2012; Renson et al., 2013), depending on the virulence the pathogenic strain the individual is exposed to. Duration of immunity is at least 6–10 months regardless of the route of administration (intramuscular or oral; Kaden and Lange, 2001). Indications exist that immunity might be even life-long. In the European Union (EU), oral vaccination of wild boar has proven to be very effective for the eradication of the virus (EFSA, 2008). The major drawback of live vaccines is that it is impossible to differentiate antibodies induced by field virus infections from antibodies induced by vaccination.

#### Marker Vaccines

Baculovirus-expressed E2 recombinant protein subunit vaccines were the first generation of non-replicative marker vaccines for CSF. The efficacy of these two available E2 sub unit vaccines was extensively studied and was determined to be lower than the efficacy of classic C-strain vaccines (Uttenthal et al., 2001). Vaccination could not prevent the “carrier sow syndrome” and subsequently the late onset of CSF (Depner et al., 2001). An additional drawback of this vaccine is that it cannot be used for oral vaccination in baits. In recent years, new approaches have been used to develop marker vaccines that allow a DIVA principle while having the advantages of live vaccines (Beer et al., 2007; Blome et al., 2013). Two promising candidates, *pestivirus* chimera “CP7_E2alf” and flc11, were then compared within an EU-funded research project to decide which would be followed up for licensing. Based on the comparative trial and pre-existing data on safety and efficacy, “CP7_E2alf” was chosen for further assessment and marketing (Blome et al., 2012).

Regarding the BVDV/CSFV chimera “CP7_E2alf,” which carries the CSFV E2 and a BVDV backbone (Reimann et al., 2004), immunization and challenge trials showed that after a single intramuscular or oral vaccination, the antibody titers were stable for a minimum of 6 months and full protection from lethal challenge infection was observed. In follow-up experiments, this vaccine proved to be safe and efficacious against challenge with CSFV strains of different genotypes and virulence. The vaccine preparation for intra-muscular use has been recently registered in the EU (Suvaxyn CSF Marker, Zoetis), and there is supportive data showing potential for oral vaccination development.

### Diagnostic Tools

For the diagnosis of CSF in wild boar and monitoring following oral vaccination, all methods used for domestic pigs may be used (Blome et al., 2006). These techniques include both direct (virus isolation, antigen detection, genome detection) and indirect (antibody) test systems. The commercial E2-ELISA displays a sensitivity that is in general quite similar to the virus neutralization test (VNT). The specificity is usually high, between 98 and >99.5%. However, cross-reactions may occur with ruminant pestiviruses, especially BDV. Moreover, poor quality of samples derived from wild boar can lead to false positive and negative reactions, especially in ELISA (EFSA, 2008). In recent years, a combination of commercial E2 antibody ELISAs and CSFV specific real-time RT-PCRs has been used to monitor wild boar populations. As CSFV does not present different serotypes, no problems in detecting antibodies against different strains are anticipated (for testing different genotypes see Schroeder et al., 2012). Suitable sample matrices are blood or serum, different organs and even swab samples (Anonymous, 2002; Petrov et al., 2014). Virus isolation in susceptible cell cultures and neutralization tests have been employed as confirmatory assays for CSFV and CSFV specific neutralizing antibodies, respectively. For C-strain vaccination scenarios, sampling and testing strategies have been developed that allow targeted testing (Kaden et al., 2006). Live attenuated vaccine strains such as the C-strain or CP7_E2alf show a very limited replication even in the target host (Koenig et al., 2007). However, highly sensitive detection techniques such as real-time RT-PCR can lead to vaccine virus detection in blood and organs from wild boars that have received oral vaccination (Blome et al., 2011). To rapidly differentiate these detections from field virus infection (genetic DIVA), specific real-time RT-PCR systems have been developed for different C-strain variants and marker vaccine CP7_E2alf (Li et al., 2007; Huang et al., 2009; Leifer et al., 2009). While traditional live attenuated vaccines do not allow a serological DIVA concept, CP7_E2alf has a marker system that is based on the detection of CSFV Erns antibodies. Animals vaccinated with CP7_E2alf will carry CSFV E2 but not CSFV Erns antibodies while field virus infected animals will also show CSFV Erns responses. At present, one Erns ELISA is commercially available (PrioCHECK CSFV Erns, Thermofisher) and additional approaches are currently under development based on either ELISA or Luminex technology (e.g., Aebischer et al., 2013; Xia et al., 2015).

### Baits

For a feasible oral immunization scheme, a suitable delivery vehicle in the form of bait is needed. Such baits need to fulfill a wide range of requirements. The most obvious requirement is the acceptance of the bait by the target species. Bait detectability (odor, color), palatability (taste), and uptake must all be considered. Wild boars are omnivores and consume a wide range of foods, but can have very clear preferences for certain food items such as acorns (Brandt et al., 2006; Ballesteros et al., 2011). During initial bait studies with wild boars kept in enclosures, no clear preference were observed between different aromas tested (e.g., apple, corn, almond, hazelnut, truﬄe, potatoes). This was also confirmed during subsequent field studies with free-ranging wild boars (Schuster, 1996). The animals tend to prefer baits containing plant-derived compounds, especially corn meal, over animal-derived compounds (Schuster, 1996). Based on these studies, the present commercial bait matrix that accompanies the Riemser Schweinepestoralvakzine (IDT Biologika, former Riemser Arzneimittel) consists of corn meal, paraffin wax, milk powder, aroma (almond), and hardened coconut oil. To assess bait uptake, bait markers can be incorporated in the bait matrix or the blister. During initial field trials, tetracycline was used (Kaden et al., 2000). However, bait markers efficient in this species (i.e., tetracycline, iophenoxic acid, rhodamine) are supposed at risk for human health, since wild boar are hunted and consumed by people (Ballesteros et al., 2013; Sage et al., 2013) and subsequently increase the overall cost of vaccination, which compromises the use of chemical markers at a large scale in natural populations (EFSA, 2008; Anses, 2012). Beef tallow which was used in the original bait, was removed from the bait matrix because regulatory requirements limited the use of certain bait materials (e.g., products derived from terrestrial animals; tissues that may transmit spongiform encephalopathy). The bait has a relatively low melting point (30°C) and is therefore not suitable for distribution in areas during periods with high elevated temperatures. To protect the liquid vaccine against environmental factors, including the bait matrix, the formulated vaccine (1.6 ml) is filled in a vaccine container after vaccine production. Subsequently, the PVC vaccine container (20 mm × 20 mm × 7 mm) is sealed with an aluminum foil and incorporated into the bait matrix (40 mm × 40 mm × 15 mm). For CSFV it is important that the vaccine is released in the oral cavity so that it can be taken up by the tonsils to initiate the immune response. Therefore, to release the vaccine in the oral cavity of the wild boar, the animal needs to perforate the vaccine container with its teeth. If baits are too small, it could be swallowed without chewing and the vaccine blister will not be perforated. However, the present bait may be too big for piglets (<4 months of age) to consume. Faust (2007) observed that piglets only played with the baits and showed an incomplete uptake. In this case, the vaccine was not released into the oral cavity, resulting in a failed vaccination attempt in juvenile wild boar. Also, shape and texture can influence bait handling and possibly result in increased vaccine spillage (e.g., dripping on the ground). Several baits composition and shapes were tested in piglets during a former European collaborative project but did not result in a better uptake in that age class in continental European countries. This may be due to the low palatability of baits in comparison with the natural food available during spring and summer when juvenile wild boars are still piglets (Sage et al., 2011; see An Adapted Bait Delivery Process). A field trial for wild boar vaccination in Italy with the new live marker vaccine “CP7_E2alf” and the classic IDT^®^ bait gave results similar to oral vaccination campaigns with C-strain which is encouraging for future vaccination applications even though the commercial version of the oral marker vaccine is not yet available (Feliziani et al., 2014).

## Deployment Challenges

### A Short History of Oral Mass Vaccination

There is always a big gap between the development of vaccination tools by researchers and the deployment of vaccination in the field (van Oirschot, 2003). First, research results have to be translated into industrial products. Then, field trials are needed to assess the efficacy of the bait delivery process (see An Adapted Bait Delivery Process and Assessing Vaccination Efficacy) and allowing an official vaccine registration (OIE [World Organisation for Animal Health], 2012). After that, the process has to be adjusted to the specific local environmental conditions (An Adapted Bait Delivery Process). Regarding the C-strain, the only available oral vaccine currently available on the market, the industrial production and field trials were mainly implemented in Germany during the 1990’s and early 2000’s by Kaden (1998), Kaden et al. (2000, 2002, 2003, 2004, 2005) and the IDT^®^ company. Once the strategy is officially adopted, many practical problems must be solved before deployment including prerequisite and exhaustive census of the vaccination grounds, organizing the logistics for frozen or cold transportation and storage of several thousands of vaccine-baits within isolated areas, delivering the technical information to hunters and controlling bait distribution and consumption in the field. Stop-hunting 1 week before and during bait distributions has been implemented to avoid animal disturbance and to limit the risk of false PCR-positive results (Louguet et al., 2005). Vaccine-baits alone are relatively cheap (around 1 euro per bait) so the cost of treating one square kilometer of forest averages 400–500 euro per year. However, significant secondary costs are associated with the management of endemically infected areas such as testing of hunter killed animals and incidentally discovered carcasses for CSF serology and virology, the compensation for carcass destruction (CSF positive carcasses to CSF virology are destroyed), the control of carcass identification and trade. During the 2000’s in France the total cost of CSF management in wild boar was estimated around 1500 euro per square kilometer of treated forest and per year. Since the early 2000’s, the use of OMV is officially supported by the European communities (Council Directive 2001/89/EC) and has been adopted in many countries as part of their emergency plan with an important proportion of success including Germany, Luxembourg, France, Slovakia, Bulgaria, and Latvia (EFSA, 2008; Pol et al., 2008) (**Table 1**). However, many challenges have to be still addressed for improving the baits delivery process, monitoring and efficacy (see further sections).

**Table 1:** Documented classical swine fever (CSF) outbreaks in wild boar in Europe and management measures including oral mass vaccination (OMV).

Period	Country and region	Reference	Infected area (max)	Vaccinated area (max)	Outbreak period	Vaccination period	Restriction period	Vaccination treatment
1992–2002	Germany, Lower Saxony	Kaden et al., 2000, 2002 FLI	6278 km^2^	1300 km^2^ (1993–1994) 5736 km^2^ (1997–2004)	12/1992 13.06.2002	10/1993 08/2004	12/1992 12/2004	Field trials Two campaigns a year
1999–2002	Germany, Saxony Anhalt	FLI	709 km^2^	3365 km^2^	12.10.1999 19.09.2000	12/1999 11/2001	12.10.1999 31.12.2002	Field trials Two campaigns a year
2001–2002	Germany, Saarland	FLI	275 km^2^	645 km^2^	26.01.2001 13.06.2002	03/2002 10/2003	01/2001 06/2004	Field trials Two campaigns a year
2002	Germany, Northrhine-Westphalia	EURL CSF-DB	759 km^2^	1531 km^2^	22.04.2002 14.10.2002	08/2002 10/2004	08/2002 09/2004	Field trials Two campaigns a year
2005–2007	Germany, Northrhine-Westphalia	EURL CSF-DB	1993 km^2^	1993 km^2^	07.10.2005 04.05.2007	12/2005 03/2010	10/2005 03/2010	Three campaigns a year
1999–2002	Germany, M-W Pomerania	Kaden et al., 2004 FLI	12928 km^2^	13942 km^2^	01.03.1993 21.07.2000	12/1994 06/2002	01.03.1993 31.12.2002	Field trials Two campaigns a year
1995–1997	Germany, Brandenburg	Kern and Lahrmann, 2000 FLI	5059 km^2^	9173 km^2^	14.03.1995 26.04.2000	04/1995 04/2001	14.03.1995 31.12.2002	Field trials Two campaigns a year
1999–2001	Germany, Baden-Württemberg	Köppel et al., 2007 FLI	703 km^2^	1291 km^2^	30.09.1998 19.11.1999	08/1999 10/2001	30.09.1998 31.12.2002	Three campaigns a year
1997–2002	Italy, Varese	Zanardi et al., 2003	370 km^2^	None	05/1997 12/2000	–	05/1997 02/2002	No OMV limited collective hunting
1985–1990	Italy, Tuscany South	Rutili et al., 1998 OIE	3800 km^2^	None	10/1985 11/1990	–	10/1985 11/1990	No OMV “Intensified” hunting
1992–1995	Italy, Tuscany North	Rutili et al., 1998 OIE	304 km^2^	None	01/04/1992 01/08/1992	–	01/04/1992 12/1995	No OMV limited collective hunting
1995–1996	Italy, Piacenza	Rutili et al., 1998 OIE	75 km^2^	None	09/1995 01/1996	–	Not documented	No OMV limited collective hunting
1998–2000	Swiss, Ticino	Schnyder et al., 2002, OIE	166 km^2^ (risk area)	No vaccination done	05/1998 01/2000	–	05/1998 01/2001 (OIE)	No OMV limited collective hunting
1999–2003	Germany, Rhineland-Palatinate, Eifel	von Rüden et al., 2008 EURL CSF-DB	8568 km^2^	8600 km^2^	05.01.1999 24.03.2003	02/2002 03/2005	01/1999 03/2008	Three vaccination campaigns a year
2002–2004	Germany Rhineland-Palatinate, Palatinate	von Rüden et al., 2008 EURL CSF-DB	4833 km^2^	4300 km^2^	23.10.1998 12.11.2004	01/2003 02/2006	06/2005 02/2008	Three vaccination campaigns a year
2009	Germany, Right-Side of the Rhine	EURL CSF-DB	5038 km^2^	5038 km^2^	01/2009 07/2009	02/2009 04/2010	01/2009 06/2012	Three vaccination campaigns a year
2009	Germany, Rhineland-Palatinate, Palatinate	EURL CSF-DB	862 km^2^	862 km^2^	02.03.2009 30.04.2009	03/2009 04/2010	02/2009 06/2012	Three vaccination campaigns a year
1992–1997	France, Vosges du Nord	Rossi et al., 2005a,b		No OMV	01/1992 01/1997	–	01/1992 12/2000	No OMV normal collective hunting
2002–2003	Luxembourg, whole country	SANCO 10257/2003, Brauer et al., 2006 EURL CSF-DB	2592 km^2^	2592 km^2^	11/2001 08/2002	03/2003 09/2005	11/2002 09/2005	Three vaccination campaigns a year
2003	France Thionville	Pol et al., 2008	200 km^2^	No OMV	04/2002 07/2002	–	04/2003 03/2005	No OMV limited collective hunting
2003–2007	France, Vosges du Nord	Pol et al., 2008; Rossi et al., 2010; Calenge and Rossi, 2014 EURL CSF-DB	2890 km^2^	2890 km^2^ (1250)	14/04/2003 01/05/2007	08/2004 06/2010	09/2004 11/2011	Three vaccination campaigns a year
2005–2008	Slovakia	EURL CSF-DB ADNS SCoFCAH	9897 km^2^	9897 km^2^	07/2004 05/2008	02/2005 11/2010	07/2004 06/2011	Three vaccination campaigns a year
2004–2009	Bulgaria,	EURL CSF-DB ADNS, WAHID SCoFCAH	35887 km^2^	35887 km^2^	05/2004 09/2009	07/2005 To date	05/2004 To date	Three vaccination campaigns a year
2007–2009	Hungary	ADNS SCoFCAH	∼4500 km^2^		01/2007 10/2009		01/2007 09/2012	No OMV
2006–2007	Romania	ADNS SCoFCAH	63247 km^2^	63247 km^2^	01/2006 11/2007	05/2007 12/2011	01/2006 09/2012	Three vaccination campaigns a year
2002	Belgium	EURL CSF-DB ADNS	743 km^2^	–	11/2002 11/2002	–	11/2002 01/2004	No OMV
2012–to date	Latvia	ADNS SCoFCAH	∼9000 km^2^	∼5000 km^2^	16.11.2012–to date (last reported case 26.03.2015)	05/2013–to date	16.11.2012–to date	Three vaccination campaigns a year

*FLI, Friedrich-Loeﬄer-Institut, Federal Research Institute for Animal Health, Germany; EURL CSF-DB, European database for Classical swine fever; OIE, world organization for animal health; SANCO, European Commission Directorate-General for Health and Consumers; ADNS, European Animal Disease Notification System; SCoFCAH, European Standing Committee on the Food Chain and Animal Health.*

### An Adapted Bait Delivery Process

Currently, bait distribution is provided by hunters (i.e., by hand delivery) on feeding grounds. Attempts to distribute baits by aircraft were completed (Kaden et al., 2002), but are not generally used, possibly due to high costs (EFSA, 2008). Furthermore, several field studies confirmed that wild boar are omnivorous and opportunistic animals that need to be pre-baited before vaccine delivery in order to limit bait uptake by non-target species (e.g., red fox, badgers, martens, birds, etc; Sage et al., 2011; Ballesteros et al., 2013). The C-strain vaccine bait has been classical delivered under ground to target wild boar specifically (Kaden et al., 2002) and to protect live-vaccine against damage due to hot temperatures and consecutive efficacy loss. However, recent behavioral studies using camera traps and different delivery process demonstrated that baits put under ground may decrease wild boar uptake (especially in juvenile boar) while not effectively preventing the consumption by non-target species (Sage et al., 2011). Low bait uptake in piglets less than 6 months old has been a constant problem in previous vaccination attempts (Sage et al., 2011) and the consecutive low vaccination rates in that age class (Rossi et al., 2011; Calenge and Rossi, 2014) have been a well know factor decreasing vaccination efficacy in both human and animal populations (Anderson and May, 1990; see Retrospective Analyses based on Hunting Data). Interestingly, using specific feeders for excluding big animals did not improved the bait uptake in piglets (Sage et al., 2010) and small baits that were efficiently consumed by piglets in Spain (Ballesteros et al., 2009) were poorly consumed in continental European areas, possibly as a result of different food availability between continental and Mediterranean ecosystems during summertime (Sage et al., 2010, 2011). The current vaccination process is based on three double campaigns in spring, summer, and autumn; each campaign comprising two vaccine-baits-distribution spaced by 28 days, aiming at maximizing antibody titters (by booster vaccination) and the proportion of vaccinated juvenile wild boars (Kaden et al., 2004; EFSA, 2008). However, recent retrospective studies, taking into account wild boar demography and spatial structure, confirmed that bait uptake in juvenile wild boar less than 1 year is always very low in summer (∼5%) and autumn (<30%) compared to spring (40–70%). This explains why 1 year is necessary for reaching a maximum seroprevalence in wild boar populations within these areas (Calenge and Rossi, 2014). The classic vaccination process corresponds to the delivery of about 40 baits per vaccination ground and a density of one to two vaccination ground per square kilometer of treated forest (EFSA, 2008). The vaccination effort and the percentage of vaccinated wild boar are correlated until an optimum (i.e., 1.25 baiting places per km^2^ in North-eastern France) but vaccination efficacy is strongly influenced by the season and year in relation to natural food competing with feed stations and baits (Calenge and Rossi, 2014). Thus, it is probable that uncontrolled factors (i.e., temperature, rain fall, population dynamics, etc) generate huge variations in the vaccination success even though the baiting process is conserved or intensified from year to year. Finally, vaccination success relies on the delimitation of the vaccinated area, which we further discuss in Sections “Assessing Vaccination Efficacy and Alternative or Complementary Strategies.”

### Monitoring CSF within Vaccinated Areas

Monitoring CSF outbreaks when vaccinating with the C-strain vaccine has been challenging during the past deployment attempts, since the non-marker vaccine (C-strain) strongly impacts the performance and significance of the diagnostic tools. First, antibodies targeting the C-strain and the wild CSFV strains cannot be differentiated using serological tests (Beer et al., 2007; Dong and Chen, 2007; Blome et al., 2013). Therefore, during OMV, seroprevalence is indicative of an average level of population immunity but not of CSF circulation (Kaden et al., 2006; Calenge and Rossi, 2014). Second, the proportion of viropositive individuals is very low in vaccinated populations, which compromises the probability of virus detection even though hunting bags are exhaustively examined within infected areas, (i.e., representing several thousands samples per year; van Oirschot, 2003; Rossi et al., 2010) using highly sensitive PCR tools (Blome et al., 2006). Additionally, among sparse PCR-positive results, false positive may occur after several vaccination campaigns, corresponding to C-train genome traces (in spleen samples), which interfere also with monitoring efficacy and justified the development of DIVA-PCR we yet described in Section “Diagnostic Tools” (Blome et al., 2011). After the completion of vaccination, seroprevalence remains high for some years, so that CSF circulation cannot be correctly monitored; surveillance has thus to be maintained for at least 3 years after OMV completion even though no more cases are detected (Kaden et al., 2006; Rossi et al., 2014; Saubusse et al., accepted). The longitudinal monitoring of capture-marked-recaptured wild boar may help in better interpreting wild boar immune response, but is also spatially limited and time consuming (Rossi et al., 2011; Saubusse et al., accepted). The future development of a new marker vaccine would help to improve outbreak monitoring within vaccinated areas, since antibodies from vaccinated and infected animals could be differentiated using companion serological tests.

## Assessing Vaccination Efficacy

### Retrospective Analyses based on Hunting Data

#### At the Outbreak Level

Vaccination success was determined through retrospective studies based on field hunting data collected in Germany, Luxembourg, and France, which showed evidence of a significant increase of seroprevalence up to 60% and a quick decrease of viroprevalence under 1% within vaccinated areas within 1–6 years (Kaden et al., 2002, 2003; von Rüden et al., 2008; Rossi et al., 2010). Nevertheless, vaccination success has not been complete as CSFV has been spreading in spite of vaccination in continuous forested areas (Kaden et al., 2002) and CSFV may re-emerge after disappearance of the virus for several years (EFSA, 2008). Such problems possibly arose because (i) vaccination areas were too small compared to the actual area at risk of disease spread (i.e., the whole connected forested areas), (ii) juvenile were not correctly immunized during the critical average age of infection, (iii) vaccination was not maintained for enough time (EFSA, 2008). Retrospective analyses performed in France highlighted that, vaccination is not necessarily preventing CSF spread within connected forested areas, due to the fact emergency vaccination is not effective enough to break the chain of transmission immediately. Nevertheless, proactive vaccination, when performed within a 24 km width buffer vaccination area surrounding the virus wave front (i.e., corresponding to 1 year virus spreading average distance), is able to limit further disease persistence possibly by preventing the re-invasion between neighboring sub-populations (Rossi et al., 2010). These results are logical since a maximum seroprevalence is ultimately reached after 1 year of deployment (i.e., after a complete cycle of three double vaccine distributions was achieved; Rossi et al., 2010). An accurate delimitation of infected and vaccinated areas, according to landscape/forest structure and existing barriers, is thus considered as a critical step for controlling CSFV in wild boar using vaccination (EFSA, 2008).

#### At the European Level

**FIGURE 1:**
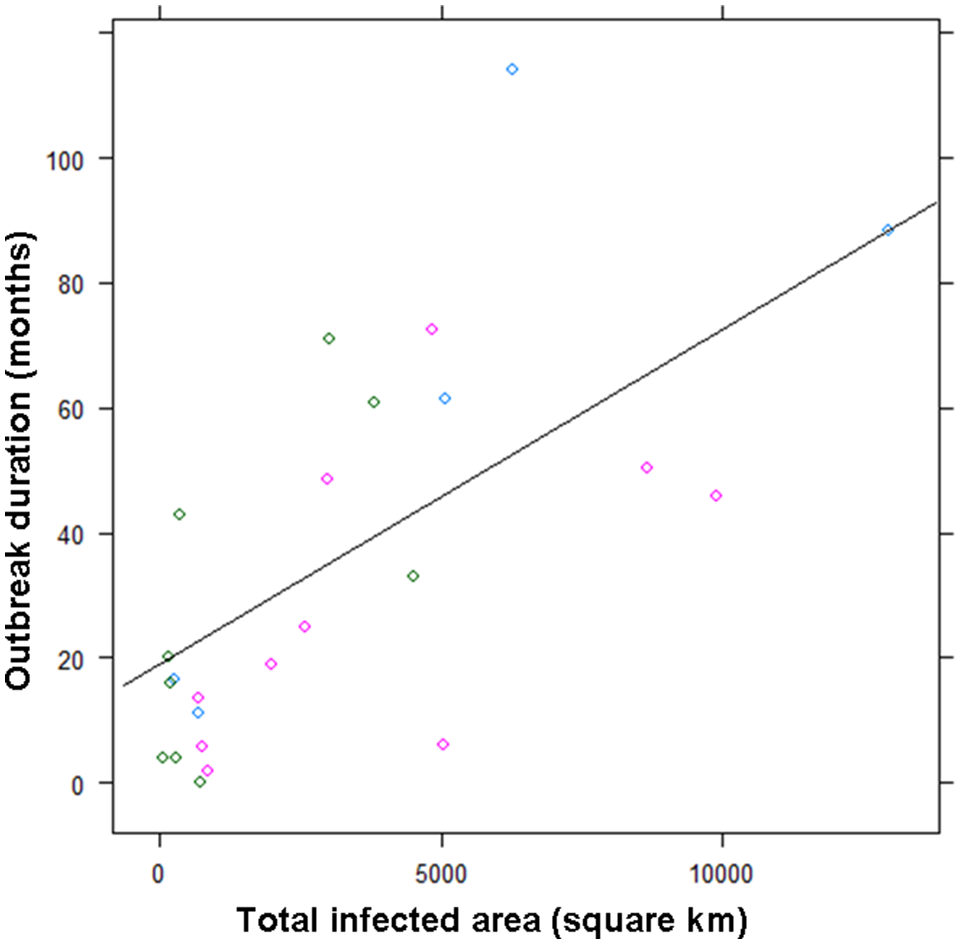
**Duration of outbreak (number of months with viropositive results) as a function of infected areas and of the vaccination treatment over 24 “fade out” outbreaks (1985 to 2009).** Green circles correspond to non-vaccinated areas, blue circles correspond to primary field trials using two simple or double campaigns a year (before 2002), pink circle correspond to the current oral mass vaccination (OMV) scheme using three double campaigns a year (mainly after 2002). The line is representing the average linear regression linking the duration of outbreak to infected areas.

As previously discussed by Rossi et al. (2005a) and Kramer-Schadt et al. (2009), the dimension of the risk areas (in square km^2^), which depends on forest extend and structure, has been the main factor influencing outbreaks duration from 1985 up to 2009 (*R*^2^ = 0.46, *p* < 0.001, **Table 1** and **Figure 1**), When OMV was performed three times a year, the average outbreaks duration decreased (OMV_effect_ = -13 months, ±10.8, *p* = 0.22), but at the same time, the average delay between the last viropositive result and the end of restrictions measures increased (OMV_effect_ = +12.9, ±5.1, *p* = 0.02); possibly as a result of the confusing effect of OMV on serological and PCR results (see Deployment Challenges). Thus, OMV has not reduced the cost of CSF management, but it has been the only strategy preventing outbreaks re-emergence in large connected forested areas in Europe [e.g., Palatinate (Ge) and Vosges du Nord (Fr)] (EFSA, 2008; **Table 1**).

### Modeling Efficacy

The epidemiological modeling of wildlife diseases is a tool used to support disease control and mitigation measures. Mathematical models from population ecology demonstrate the principle of OMV in wildlife (Anderson et al., 1981). The approach focuses the estimation of a minimum population proportions that should be protected against infection during an OMV program to halt the spread of CSF. Along with early field trials of OMV such models proposed an average 40–50% population level immunity as sufficient to stop CSF spread (Hone et al., 1992; Guberti et al., 1998). However, the models relied on critical simplifications that may have led to an underestimation of the threshold population immunity. The infectious period may differ between infected wild boar individuals because immunocompetency varies according to age and body condition, and the occurrence of rare chronic (i.e., long-lasting) infection is a critical factor regarding CSF dynamics (Kramer-Schadt et al., 2009) and vaccination efficacy (Lange et al., 2012), which could not be caught by average simplest models. Moreover, most of the wild boar populations subjected to OMV are big and distributed over large connected areal (Rossi et al., 2005a). Thus, the assumption of sufficiently contact within the complete population on the temporal scale of an individual CSF infection was not biologically reasonable and spatially explicit models were required for better understanding the persistence patterns of CSF in the wild. Next step to support OMV planning was the application of stochastic meta-population modeling that suggested a useful population level immunity of 60% (EFSA, 2008) in line with field estimates from vaccination areas. More recent research, implementing individual-based models of wild-boars moving and getting infected in a spatially explicit habitat landscape, were finally implemented for testing different vaccination strategies (Lange et al., 2012). These last models highlighted that the probability of CSFV eradication particularly relied on the implementation of preventive vaccination and the maintenance of vaccination effort for at least 5 years (Lange et al., 2012). A possible next modeling step could be to take into account the temporal and spatial variation of vaccination efficacy (Calenge and Rossi, 2014).

### Virus Evolution under Vaccination Pressure

Classical swine fever virus can be assigned to three genotypes with three to four sub-genotypes each. These genotypes do not translate into serotypes that pose a problem in diagnostics or vaccination. Over the last decade, mainly strains of genotype 2, especially 2.1 and 2.3 were circulating in Europe. In the wild boar, only subtype 2.3 was prevalent (for review see Beer et al., 2015). In general, CSFV is exceptionally stable for an RNA virus (Vanderhallen et al., 1999), and mass application of the C-strain did not induce a detectable evolution of the virus in the wild (see below). Indeed, due to this observed genetic stability, even a single point mutation could be considered relevant for molecular epidemiological studies of CSF outbreaks. The evidence of separate evolution of the two outbreaks in the 1990–2000’s traced one outbreak to the strain Rostock and the other to the strain Uelzen in France and Germany. This has shown that environmental factors including absence of a forest continuum between two regions have a real contribution to containment of the disease (Pol et al., 2008; Simon et al., 2013). More recently, full genome sequencing has been carried out to investigate the evolution of the CSFV during a long-term outbreak within the wild boar population in the Vosges du Nord mountains region. The samples were chosen based on the results of partial sequencing (Simon et al., 2013) and the availability of temporal and spatial data in relation to the application of the C strain vaccine. It was demonstrated that the identified clusters were associated with the presence of barriers including roads, rivers, or railways rather than to a viral strategy to escape to the vaccine immune response.

## Alternative or Complementary Strategies

### What about the Depopulation Option?

During the 1980’s and early 1990’s, the “pre-vaccination era,” wild boar density was considered to be the main factor favoring virus emergence and endemic persistence; reaching a threshold value of about 1 wild boar per km^2^ was recommended for achieving virus eradication according to a pure density-dependent argument and assuming a high virulence of virus strains (Hone et al., 1992; Guberti et al., 1998). At that time, CSF control in wild boar was supposed to be achieved through depopulation only, such as used in domestic pigs, and depopulation was expected to be performed by increasing hunting pressure and/or destroying trap-captured animals (EFSA, 2008). Such depopulation strategy was even recommended by the European experts and the former EU legislation to the member states faced with CSF in wild boar (Alexandrov et al., 2011; 91/685/CEE, Art. 6, par. 5, letter e). In practice, the depopulation strategy has never been satisfactory for controlling outbreaks in wild populations, and was even considered as an aggravating factor for CSF spread and persistence by some authors (Laddomada, 2000; Artois et al., 2002; Schnyder et al., 2002). Many reasons could explain the failure of the depopulation strategy, even if the density-dependent approach had been effective, including (i) wild boar density at which the virus could fade out was probably lower than that which could be achieved through hunting, (ii) the exact population size and density of the involved wild boar population were rarely known, and (iii) the low acceptability of depopulation among hunters, especially when targeting females and very young piglets (EFSA, 2008). In the field, it is likely that the infected populations were managed according to typical hunting strategies, focused on maintaining or increasing a populations’ size, with moderate hunting pressure on reproducing females (Gamelon et al., 2012; Keuling et al., 2013). One may even fear that the actual hunting pressure during the early stage of the outbreaks was actually lower than before CSFV emergence due to the lethality induced by the virus and the difficulty of hunting sparse animals (Rossi et al., 2005a). Additional “depopulation tools” such as trap-capture or poisoning, were sometimes carried out in the field in Europe (Alexandrov et al., 2011), but trapping is not cost-effective for the large-scale management of wild boar and poisoning has been considered unacceptable for both animal welfare and human safety in Europe (EFSA, 2008). Finally, more recent studies suggested that the density-dependent approach was not effective for eradication of CSF given that: (i) wild boar density is not the main factor driving CSF persistence which rather relys on landscape structure (related to the total population size at risk) and the moderate virulence of virus strains involved in wild outbreaks (Rossi et al., 2005a; Kramer-Schadt et al., 2009), (ii) increasing hunting pressure might increase population turnover and increase the risk of disease persistence in naïve piglets (EFSA, 2008), (iii) hunting is known to increase home range size and could thus contribute to increasing the mixing and disease transmission between social groups or subpopulations (Keuling et al., 2008; Saïd et al., 2012). It is notable that the depopulation strategy was again addressed by the European communities regarding the management of African Swine Fever (ASF) recently emerging in the European wild boar; depopulation was not considered as an suitable option given its lack of efficacy, in spite of a lack of available vaccine (EFSA, 2014; Gavier-Widén et al., 2015).

### Restraining Wild Boar Movements and CSF Spread

The intrinsic spreading of CSF within natural wild boar populations relies mainly on the forest structure and the presence of physical barriers. Due to the forest habitat of the species, the main factor influencing CSF spread (and persistence) within wild boar populations is the connectivity (∼distance) between neighbor forest patches (Rossi et al., 2005a, 2010). Physical barriers may also participate in limiting animal movements, especially fenced motorways and major rivers or lakes (Laddomada, 2000; Schnyder et al., 2002; Rossi et al., 2010). However, the efficacy of barriers for preventing animal movements depends on their nature and/or the combination with the forest structure (Martin et al., 2013). The reliability and practicability of erecting fences for preventing disease spread in wild boar has been addressed regarding both CSF and ASF control in Europe (EFSA, 2008, 2014). In theory, this solution is attractive especially when OMV is not possible, but in practice it has been found poorly satisfactory (e.g., the recent spread of ASF in Lithuania in spite of huge fencing efforts, EFSA, 2014). The main problems with using fences include that: (i) it is costly, (ii) it takes time to build during which diseases may spread further, (iii) our knowledge about the exact position of the wave front of a wildlife disease at time t is not always accurate, (iv) wild boar are very good at damaging fences and fences must be regularly checked and fixed, which is costly and seldom achieved. As discussed previously, collective hunting is expected to increase animal home range and dispersal, thus hunting bans or banning of hunting dogs during collective hunting has been implemented around physical barriers to limit the risk disease spread out of infected areas (Louguet et al., 2005; Rossi et al., 2011). Nevertheless, hunting restrictions do not prevent natural seasonal movements of wild boars, which are often unrelated to human activities (Siat et al., 2010). Finally, a main aspect for control of CSF spread in wild boar is the prevention of direct and indirect contacts between wild boar and domestic pigs, which relies essentially on (i) biosecurity measures and swill feeding control at pig farms (ii) control of wild boar feeding, carcass trade and viscera releases (Laddomada, 2000; EFSA, 2008).

## Conclusion

Wild boar vaccination against CSF has been applied for more than 15 years in the EU using a highly efficient live attenuated vaccine, the C strain-Riems, delivered in baits. While intensifying hunting or erecting fences has not been adequate for preventing disease spread or persistence, OMV has proved to be effective in maintaining herd immunity and achieving CSF control; it is the only available method for CSF eradication in large forested areas. On the other hand, CSF may also be quickly eradicated without vaccination in small forested areas (<1000 km^2^) well delimited by physical barriers by establishing hunting restrictions to avoid disease spread (e.g., Thionville in France and Ticino in Switzerland). Obviously, CSF control is also dependant on the precautionary measures taken for carcasses control and pig farm biosecurity. An integrated strategy is preferred to a single one to maximize the chance of success and also combining other strategies with vaccination should be considered. It is interesting to note that intensified hunting, feeding bans and fencing were recently re-evaluated as possible management measures for controlling ASF in Europe and were not considered to be adequate since now, given past experience during CSF outbreaks (EFSA, 2008, 2014).

The current OMV method relies on multiple bait distributions per year which represents a huge collective effort. Thus, it relies on the involvement of the stakeholders including hunters, wildlife agencies, local, and central veterinary services, and local and reference laboratories. Efforts must be coordinated between neighboring regions or countries when sharing the same forested areas, wild boar populations and outbreaks. Management success relies not only on baiting intensity or the number of vaccination campaigns. First, the landscape structure (forest and barriers) has to be considered for determination of the infected areas and development of a monitoring scheme. This enables quick and proactive deployment of OMV (24 km buffer area). Second, a multiple-year application of OMV is necessary to prevent CSF re-emergence. Furthermore, OMV does not generate genetic evolution of the virus strains.

Oral mass vaccination is costly during and after OMV deployment; carcass monitoring and restrictions last for several years after vaccination due to the confounding effect of non-marker vaccine on surveillance hunting data. Diagnostic tools have to be reliable and adapted for this purpose. Future outbreaks could be addressed and controlled more rapidly using oral marker vaccine (which is validated but not yet commercially available) and companion serological tools (which have to be validated). Even if the youngest piglets cannot eat the baits, experience of the last years of vaccination showed that they were protected by maternally derived antibodies. Therefore, applying repeated vaccination for adult females could avoid this possible failure in vaccination programs.

## Conflict of Interest Statement

The authors declare that the research was conducted in the absence of any commercial or financial relationships that could be construed as a potential conflict of interest.
